# The Paradox of Self-Fertile Varieties in the Context of Self-Incompatible Genotypes in Olive

**DOI:** 10.3389/fpls.2019.00725

**Published:** 2019-06-26

**Authors:** F. Alagna, M. E. Caceres, S. Pandolfi, S. Collani, S. Mousavi, R. Mariotti, N. G. M. Cultrera, L. Baldoni, G. Barcaccia

**Affiliations:** ^1^Dipartimento Tecnologie Energetiche (DTE), Centro Ricerche Trisaia, ENEA Agenzia nazionale per le nuove tecnologie, l’energia e lo sviluppo economico sostenibile, Rotondella, Italy; ^2^Dipartimento di Scienze Bio Agroalimentari (DiSBA), Istituto di Bioscienze e Biorisorse (IBBR), Consiglio Nazionale Delle Ricerche (CNR), Perugia, Italy; ^3^Department of Plant Physiology, Umeå Plant Science Centre, Umeå University, Umeå, Sweden; ^4^Laboratorio di Genomica, Dipartimento di Agronomia, Animali, Alimenti, Risorse naturali e Ambiente (DAFNAE), Università di Padova, Legnaro, Italy

**Keywords:** *Olea europaea* L., pseudo-self-compatibility, pollen-pistil interaction, sporophytic system, self-incompatibility

## Abstract

Olive, representing one of the most important fruit crops of the Mediterranean area, is characterized by a general low fruit yield, due to numerous constraints, including alternate bearing, low flower viability, male-sterility, inter-incompatibility, and self-incompatibility (SI). Early efforts to clarify the genetic control of SI in olive gave conflicting results, and only recently, the genetic control of SI has been disclosed, revealing that olive possesses an unconventional homomorphic sporophytic diallelic system of SI, dissimilar from other described plants. This system, characterized by the presence of two SI groups, prevents self-fertilization and regulates inter-compatibility between cultivars, such that cultivars bearing the same incompatibility group are incompatible. Despite the presence of a functional SI, some varieties, in particular conditions, are able to set seeds following self-fertilization, a mechanism known as pseudo-self-compatibility (PSC), as widely reported in previous literature. Here, we summarize the results of previous works on SI in olive, particularly focusing on the occurrence of self-fertility, and offer a new perspective in view of the recent elucidation of the genetic architecture of the SI system in olive. Recent advances in research aimed at unraveling the molecular bases of SI and its breakdown in olive are also presented. The clarification of these mechanisms may have a huge impact on orchard management and will provide fundamental information for the future of olive breeding programs.

## Introduction

Olive (*Olea europaea* L.) is a perennial diploid species mainly clonally propagated and diffused in the Mediterranean area as one of the oldest tree crops (Loumou and Giourga, 2003; McKey et al., 2010; Zohary et al., 2012; Mousavi et al., 2017). As in many other allochthonous, hermaphrodite, wind-pollinated species, olive is characterized by a plentiful flowering, followed by a poor fruit set, which results in low yields (Cuevas and Polito, 2004; Ben Dhiab et al., 2017; Kassa et al., 2019). Environmental conditions, such as temperature, rainfall, and wind, may strongly affect flowering time, flowering intensity, and fertilization (Fernandez-Escobar et al., 2008; Haberman et al., 2017; Benlloch-González et al., 2018). Efficient pollination depends on many factors, such as the presence of exogenous compatible pollen, the duration of stigma receptivity, the number of pollen grains, pollen-ovule ratio, and stigma morphology (Cruden, 2000; García-Mozo et al., 2004; Pinillos and Cuevas, 2009; Rojo et al., 2016). Despite the importance of these factors, the main constraints responsible for the low fruit setting of olive are undoubtedly self-incompatibility (SI) and a high percentage of ovary abortion of some cultivars (Reale et al., 2009; Seifi et al., 2015).

Self-incompatibility is one of the most effective systems adopted by flowering plants to prevent inbreeding and maintain a high diversity within species (Ferrer and Good, 2012). Within the sporophytic (SSI) and gametophytic (GSI) categories, several incompatibility systems have been reported, but only three of them have been characterized at molecular level, one for SSI (Brassicaceae) and two for GSI (one in Solanaceae, Plantaginaceae, and Rosaceae, and one for Papaveraceae) (Higashiyama, 2010). The recent discovery of the diallelic SSI system in olive and other related genera has provided evidence on the SI system operating in the Oleaceae family. In particular, it has been clarified that the inhibition of pollen tube growth takes place at the stigma, and it has been established that there are only two incompatibility groups, i.e., diallelic SI, such that olive cultivars are incompatible within groups and compatible between groups (Saumitou-Laprade et al., 2017a).

In light of this evidence, it has become even more difficult to explain the self-fertility of some varieties, which has been confirmed by numerous studies (Seifi et al., 2012; Selak et al., 2014a; Breton et al., 2016; Marchese et al., 2016a). It is also now clear that pseudo-self-compatibility (PSC)—the failure to reject self-pollen despite the presence of a functional SI system—may occur in olive, and all previous reports on olive self-fertility need to be re-interpreted or ignored when not confirmed by paternity tests of seeds deriving from selfing. In this review, we hope to shed some light on the complexity of SI system in olive, particularly focusing on the evidence for PSC, providing a synthesis of the fragmented work on this topic and a new perspective on SI in olive, supported by experimental work from our laboratory.

## Olive Self-Incompatibility

For a long time, olive has been erroneously classified as a GSI species, based on morphological traits shared with taxa manifesting a GSI system, such as wet-type stigma and bi-nucleate pollen (Serrano et al., 2008); however, only a few cytological studies supported the occurrence of this kind of SI system (Serrano et al., 2010). Recently, it has been demonstrated that the GSI model fails to explain the presence of reciprocal differences in fruit set in one-third of mates (Breton et al., 2014; Farinelli et al., 2018). This evidence, together with others, such as the inhibition of germination at the stigma and the failure to identify genes controlling GSI in olive (Collani et al., 2012), led to the hypothesis that olive may have a SSI system. The wide methodical genetic study carried out by Saumitou-Laprade et al. (2017a,b) definitively confirmed that olive has a sporophytic homomorphic diallelic system, similar to that identified in close species, such as *Phillyrea angustifolia* and *Fraxinus ornus* (Saumitou-Laprade et al., 2010; Vernet et al., 2016) and different from any other described in other plant families. The SSI system has been classified as diallelic because controlled by a single locus with only two alleles (S1 and S2), according to the segregation analysis of SI trait in a cross population (Saumitou-Laprade et al., 2017a). Thus, only two SI genotypes have been found so far, and olive cultivars can be classified, based on their belonging to the SI group, as G1 = S1S2 or G2 = S1S1. First evidence on the olive diallelic SSI system indicated that it is not accompanied by heterostyly (Saumitou-Laprade et al., 2017a); however, dedicated studies to confirm this preliminary data were not conducted.

**Figure 1:**
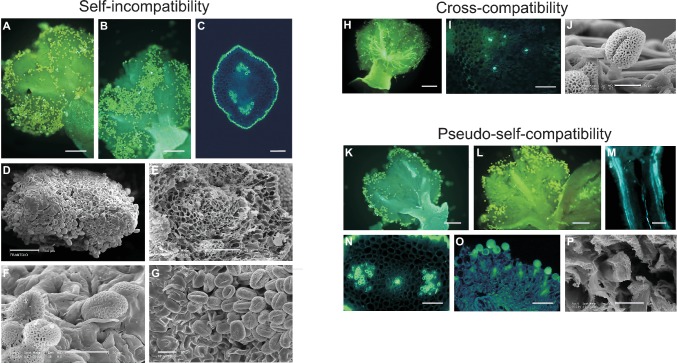
Self-incompatibility, cross-compatibility, and pseudo-self-compatibility in olive. **(A–G)** Self-incompatibility reaction in flowers of cultivars Frantoio, Leccino, and Dolce Agogia at 6 and 15 days after anthesis, as observed by epifluorescence images after aniline blue staining **(A–C)** and scanning electron microscope (SEM) images **(D–G)**. Pistils of cv. Frantoio **(A,B)** and transversal sections of style of cv. Leccino **(C)** and cv. Dolce Agogia **(E)** showing the absence of pollen tube growth in the style. Stigma of cv. Frantoio **(D,G)** and cv. Leccino **(F)** showing the absence of germinated pollen grains into the style. Bars = 150 μm **(A,B)**, 100 μm **(D)**, 50 μm **(C,E)**, and 20 μm **(F,G)**. **(H–J)** Cross-compatibility of cv. Frantoio (incompatibility group G1), pollinated with compatible G2 pollen, as observed by epifluorescence images after aniline blue staining **(H,I)** and SEM image **(J)**. Cross-pollination at 72 h after anthesis showing pollen-pistil compatibility **(H)**. Transversal section of style showing pollen tube growth throughout the pistil **(I)**. Stigma showing germinated pollen grains **(J)**. Bars = 150 μm **(H)**, 50 μm **(I)**, and 10 μm **(J)**. **(K–P)**: Pseudo-self-compatibility in self-pollinated flowers, as observed by epifluorescence images after aniline blue staining **(K–O)** and SEM image **(P)**. Flowers were collected at 6 **(M–P)** and 15 **(K,L)** days after anthesis. Pistils showing pollen tube growth in the style **(K–M,O)**. Transversal sections of style showing pollen tube growth through the stylar tissues (**N,P**). Bars = 150 μm **(K–M)**, 50 μm **(N–O)**, and 6 μm **(P)**.

The general behavior of pollen within pistil tissues, under self- or incompatible cross-pollination, is represented in Figure 1. After self-pollination (Figure 1, panels A–G), most of the pollen grains landing on the stigmatic surface do not germinate, and others start germinating but do not penetrate nor grow into the transmitting tissue of the style, indicating that inhibition of pollen tube growth takes place at stigmatic level, according to the sporophytic nature of the olive SI. By contrast, during cross-pollination with compatible pollen (Figure 1, panels H–J), while a high number of pollen grains germinate on the stigma and grow toward the style, a few of them are also able to penetrate the transmitting tissue of the style and reach the ovule. Generally, only one pollen tube (and rarely two or three) grow toward the ovary and reach the carpels to penetrate an ovule (Ateyyeh et al., 2000; Seifi et al., 2011).

It has also been observed that the SI group or the genotype of the pollen recipient may have a significant effect on the length that pollen tubes may reach in the stigma prior to being arrested due to the SI reaction. In general, in G1 plants, pollen grains do not germinate at all or tube growth stops shortly after germination, whereas a higher variability can be observed in G2 plants, characterized by short or long pollen tube growth (Saumitou-Laprade et al., 2017a). This behavior, which probably contributed to the earlier difficulties in the classification of olive as GSI or SSI species, might be explained with a different timing of the SI response after the recognition of self-pollen.

## Olive Pseudo-Self-Compatibility

The current knowledge on olive SI system indicates that all olive cultivars are self-incompatible (Saumitou-Laprade et al., 2017a); however, some of them can behave as PSC in particular conditions (probably due to both genetic and environmental factors), being able to overcome SI and produce seeds after self-pollination (Breton et al., 2016; Marchese et al., 2016a; Saumitou-Laprade et al., 2017a). For a long time, olive cultivars were classified into self-compatible and self-incompatible cultivars, with most of them considered as self-incompatible (Wu et al., 2002; Mookerjee et al., 2005; Seifi et al., 2011; Sánchez-Estrada and Cuevas, 2018). Self-compatibility tests carried out in several studies have shown a low but certain rate of self-fertilizing cultivars, that were, consequently, considered to be partially self-compatible (Androulakis and Loupassaki, 1990; Ateyyeh et al., 2000; Moutier, 2002; Kasasbeh et al., 2005; Selak et al., 2011; Taslimpour and Aslmoshtaghi, 2013; Breton et al., 2014; Koubouris et al., 2014; Farinelli et al., 2018). Unfortunately, due to the lack of molecular tests to assess the origin of putatively selfed seeds and the use of unreliable materials and protocols for cross- and self-pollination experiments, some varieties were erroneously considered as self-compatible and were later proved totally self-incompatible. As an example, cv. Arbequina, previously classified as self-compatible (Cuevas, 2005), was then shown as self-incompatible (Díaz et al., 2006; Marchese et al., 2016b). Similarly, as a probable consequence of the multiple factors affecting self-fertilization success, some varieties were considered self-incompatible, as cv. Koroneiki (Mookerjee et al., 2005; Seifi et al., 2011), that later on definitively showed a low but reliable self-fertilization rate (Marchese et al., 2016b). Differences were also found among clones of the same cultivar, as in the case of cv. Leccino, mostly resulting in self-incompatible (de la Rosa et al., 2003), but with some clones partially self-compatible (Solfanelli et al., 2006).

Based on the recent evidence about the SI system operating in olive, the cultivars considered representative of the Mediterranean cultivated olive diversity showed a clear self-incompatibility reaction and inhibition of self-pollen growth (Saumitou-Laprade et al., 2017a). These results definitively support that the genetic architecture of olive SI excludes, in theory, any form of self-fertilization. The contrasting evidence that some cultivars may produce selfed progeny hints that a mechanism of incompatibility breakdown exists and that this mechanism is presently still unknown. In view of these findings, we can now conclude that olive shows PSC. This behavior can explain the contradictory data on the self-compatibility tests, and it is in agreement with previous literature reporting that the percent of successful self-fertilization was significantly lower than the fertilization rate under open- or cross-pollination (Griggs et al., 1975; Ateyyeh et al., 2000; Kasasbeh et al., 2005; Mookerjee et al., 2005). The production of truly self-seedlings has been confirmed by numerous studies where paternity tests have been applied (de la Rosa et al., 2004; Mookerjee et al., 2005; Marchese et al., 2016b).

PSC pollen growth reaction seems completely different from the compatibility response observed under pollination with compatible pollen. In self-pollinated pistils of cv. Frantoio, in fact, in most cases, pollen grains do not germinate at all, even after 6 and 15 days after flower opening and pollen-stigma contact (Figure 1, panels K and L), but in few cases, one or few pollen tubes may grow into the style and likely reach the ovary (Figure 1, panels M–P).

## Factors Affecting the Pseudo-Self-Compatibility

Pseudo-self-compatibility has been observed in numerous self-incompatible species in Asteraceae, Brassicaceae, Fabaceae, Poaceae, Ranunculaceae, Solanaceae, and other families (Good-Avila et al., 2008; Crawford et al., 2015; Liao et al., 2016). In these species, numerous external and internal factors seem to affect the ability of plants to overcome SI barrier, including the pollen germination speed, the relative growth rate of self-pollen tubes compared to cross-pollen grains, and the flower aging (Levin, 1996; Stephenson et al., 2000; Good-Avila et al., 2008; Horisaki and Niikura, 2008). The factors affecting the overcome of olive SI system are not yet known, but the available data indicate that both genotypic and environmental factors can play a role in this process. By contrast, flower age does not seem to affect PSC, considering that it has been observed in stigmas of all developmental stages.

### Environmental Factors

It is well established that environmental conditions may affect self-fertility of self-incompatible plants. In particular, SI can be overcome by high temperatures (Okazaki and Hinata, 1987; Wilkins and Thorogood, 1992; Horisaki and Niikura, 2008), high humidity (Ockendon, 1978), and chemical treatments (Lao et al., 2014; Yang et al., 2018). Also, different environments and artificial pollination techniques may favor self-fertility (Do Canto et al., 2016).

In accordance with these studies, it has been reported that environmental conditions may affect the SI reaction and fruit set in olive, and, among them, temperature appears to play a key role (Orlandi et al., 2010; Selak et al., 2013; Haberman et al., 2017). It is thought, in fact, that SI in olive is temperature-dependent (Suárez et al., 2012), and generally, high temperatures during flowering may reduce self-fertilization rate (Ayerza and Coates, 2004; Selak et al., 2013). However, the effect of temperature seems strongly genotype-dependent (Griggs et al., 1975; Koubouris et al., 2009; Selak et al., 2013). Furthermore, temperature variations strongly influence the ability of olive pollen to grow and germinate (Koubouris et al., 2009), as well as it influences stigma receptivity and ovule longevity (Selak et al., 2014b), thus affecting the fruit set (Cuevas et al., 1994).

According to the role of environmental factors in the PSC expression, results from studies on self-fertilization of olive cultivars varied particularly among different environmental conditions. For instance, variability in the self-fertilization rate has been observed among different experimental years (Griggs et al., 1975; Cuevas et al., 2001; Lavee et al., 2002; Solfanelli et al., 2006; Selak et al., 2011), orchard location (Selak et al., 2011), or different conditions, as those determined by the use of polyethylene cages (Selak et al., 2013). However, considering the high number of environmental factors changing among location and years, the available data do not allow to identify, with the exception of temperature, other key environmental factors affecting self-fertility.

### Genotype-Dependent Factors

In addition to the environmental effects, PSC appears to be strongly influenced by olive genotype, considering that self-fertilization has been exclusively observed in specific olive varieties and never reported in others. For example, successful self-fertilization in cv. Frantoio has been reported in numerous studies (Kasasbeh et al., 2005; Farinelli et al., 2008; Spinardi and Bassi, 2012; Breton et al., 2014), despite a functional SI system is also present in this variety.

**Figure 2:**
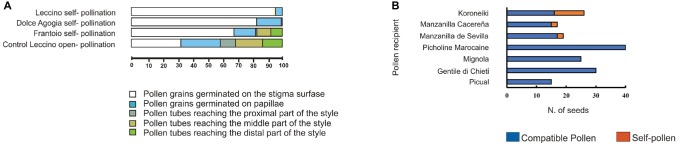
Pseudo-self-compatibility data in olive cultivars. **(A)** Variation of pseudo-self-compatibility of three olive genotypes observed under field conditions. Percentage of pollen tubes germinated in different pistil tissues of cv. Leccino and Frantoio (G1 incompatibility group) and cv. Dolce Agogia (G2 incompatibility group) was determined analyzing a total of 34.000 pollen grains. Samples were collected at 6 days after anthesis in both self-pollinated and open-pollinated (control) flowers. Pollen grains were divided into five different classes (different colors). About 9.5% of cv. Frantoio self-pollen tubes grew through the transmitting tissues reaching the distal part of the style, indicating pseudo-self-compatibility. The few pollen grains of cv. Dolce Agogia, reaching the proximal part of the style, are presumably due to slight differences in the incompatibility response of the G2 incompatibility group compared to G1. **(B)** The number of seeds obtained from pollination with compatible and self-pollen in seven cultivars under field conditions. Paternity was validated by using microsatellite markers.

Our histological and molecular study confirmed the ability of cv. Frantoio to overcome SI. By contrast, PSC was not observed for other varieties, such as cvs. Leccino and Dolce Agogia (Figure 2A), in agreement with other authors (Spinardi and Bassi, 2012; Farinelli et al., 2018). According to these data, paternity tests with microsatellite markers performed on seeds of cv. Koroneiki, Manzanilla, Cacereña, and Manzanilla de Sevilla obtained by self-pollination (Figure 2B), confirmed the origin of zygotic embryos by effective self-fertilization (Saumitou-Laprade et al., 2017a). These results validated the ability of some olive cultivars to overcome the SI barrier and confirm the occurrence of PSC.

Similar to olive, a significant difference in PSC among genotypes has been reported in other plants (Foster and Wright, 1970; Elgersma et al., 1989; Brennan et al., 2005; Baldwin and Schoen, 2017). According to the quantitative nature of PSC, this trait is typically polygenic (Do Canto et al., 2016). In some Brassicaceae species, variation in PSC has been shown to be caused by genetic variation in genes unlinked to the S-locus but involved in the SI signaling cascade that mediates the rejection of self-pollen (Liu et al., 2007; Baldwin and Schoen, 2017). This pattern has also been confirmed in either GSI or SSI species (Good-Avila and Stephenson, 2002; Mable et al., 2005; Mena-Ali and Stephenson, 2007; Crawford et al., 2015; Liao et al., 2016).

The hypothesized architecture of the S-locus in olive (G1 = S1S2 and G2 = S1S1), which guarantees a perfect 1:1 balance between the two groups (Saumitou-Laprade et al., 2017a), implies that only out-crossing between the two groups could preserve these two unique combinations, while the selfing of the heterozygous group would result in the appearance of the homozygous combination S2S2, which would lead to the imbalance of the populations in favor of one group with respect to the other. Furthermore, first evidence demonstrates that cross-pollination between self-fertile varieties and genotypes belonging to the same SI group never occurs (Saumitou-Laprade et al., 2017a). This complex picture of the paradoxical occurrence of PSC in olive is difficult to explain, also assuming the presence of a “leaky S-allele,” as observed in other plant species (Baldwin and Schoen, 2017).

## Toward the Identification of Molecular Determinants of Self-Incompatibility and Pseudo-Self-Compatibility

Further studies should be carried out, either at microscopic, genomic, and genetic levels, in order to understand the mechanisms underpinning the PSC in olive, although, it is also crucial to increase the knowledge of the molecular events occurring during the SI reaction which remain still unexplored. Studies aimed at identifying female and male determinants of the olive SI system, based on gene similarity to other plant species in which SI was molecularly characterized, have been conducted, but results of these studies indicated that olive flowers do not possess or express genes similar to the GSI determinants identified in other plants (Collani, 2012; Collani et al., 2012). On the contrary, candidate genes for female (*OeSRK-like* and *OeSLG-like*) and male (*OeSCR-like*) determinants, as orthologous of the genes that control the SSI system in the Brassicaceae family, have been cloned and characterized in olive varieties (Collani, 2012; Collani et al., 2012), and gene expression studies showed that the *OeSRK-like* gene is preferentially expressed in pistils at early flowering stages of cv. Leccino and lowly expressed in pistils at later flowering stages of cv. Frantoio, while *OeSCR-like* was found specifically expressed in dehiscent anthers of both cultivars (Collani, 2012). Despite such positive initial evidences, further genetic and molecular findings clearly demonstrated that these genes do not encode for the genetic determinants of the olive S-locus, as independent segregation of *OeSRK-like* and *OeSCR-like* genes was documented by means of SNP-based markers and no interaction between *OeSRK-like* and *OeSCR-like* proteins was observed by Yeast-2-Hybrid screens (data not shown). Although the role of these genes remains to be disclosed, our negative results corroborate an olive SI system whose genetic determinism is different from that active in Brassicaceae and from the others molecularly characterized, according to the peculiar features of olive SSI. This result supports the hypothesis that independent evolution of multiple SI systems has occurred in flowering plants (Ferrer and Good, 2012).

Activity and localization of enzymes, such as RNases, putatively involved in pollen rejection mechanism under self-pollination, have been described in olive (Serrano and Olmedilla, 2012), as well as the occurrence of programmed cell death in olive pollen, as a consequence of the SI response and the differential level of reactive oxygen and nitrogen species between self-compatible and self-incompatible pollen grains (Serrano et al., 2012). However, their role in the SI mechanism needs to be further investigated.

A high throughput transcriptomic study of olive self-pollinated flowers identified a wide set of transcripts showing extensive expression differences between pseudo-self-compatible (cv. Frantoio) and self-incompatible (cv. Leccino) cultivars, confirming that biochemical, physiological, and signaling changes occur when incompatibility is broken down (Alagna et al., 2016). These data represent a valuable resource for the identification of genes related to PSC.

In addition, several enzymes putatively involved in the regulation of pollen tube growth and in the modulation of temperature-dependent reproductive processes were identified by studying the proteomic profile of olive stigma exudate (Rejón et al., 2013). The ReprOlive database, built on the transcriptomic information of olive reproductive tissues (Carmona et al., 2015), as well as the availability of the sequence of cultivated and wild olive genomes (Cruz et al., 2016; Unver et al., 2017), provides further high valuable tools for the identification of candidate genes involved in SI signaling and for the discovery of the molecular mechanisms involved in PSC. These studies will be facilitated by the trans-generic functional homology of olive SI, which will allow for the application of discoveries from *P. angustifolia* and *F. ornus* species to olive.

## Conclusions and Perspectives

The important advances made in the study of the olive SI system do not explain the occurrence of self-fertility in some cultivars, confirmed by many studies and certainly regulated by both genetic and environmental factors. New observations should be carried out in order to clarify how germination of pollen grains and their growth within the transmitting tissue of the style may occur in a context of incompatibility. The availability of genetic materials and microscopic, genomic, and transcriptomic resources for the study of olive reproductive constrains should allow the elucidation of the selfing mechanism, as well as the identification of putative genes involved in the PSC. Understanding the mechanisms regulating SI and PSC will have a huge impact on olive orchard management and breeding programs, offering new tangible opportunities for improving olive and olive oil production.

## Author Contributions

LB and GB conceived the study. FA wrote the first draft of the manuscript. MEC and SP performed the histological observations. All the authors contributed to the content of the manuscript and revised the manuscript. All the authors agreed on the final version of this review.

### Conflict of Interest Statement

The authors declare that the research was conducted in the absence of any commercial or financial relationships that could be construed as a potential conflict of interest.
